# The positive side of hunger

**DOI:** 10.1038/s44319-026-00883-5

**Published:** 2026-07-18

**Authors:** Ľubomír Tomáška

**Affiliations:** Department of Genetics, Comenius University Bratislava, Faculty of Natural Sciences, Ilkovičova 6, 842 15 Bratislava, Slovakia

**Keywords:** Economics, Law & Politics, History & Philosophy of Science

## Abstract

A reflection on how scarcity may shape intellectual discipline and ingenuity in research, and whether today’s easy access to resources may sometimes undermine their thoughtful use.

**Figure:**
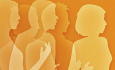

I was a student during the mid-1980s in Czechoslovakia, an Eastern European country behind the iron curtain. Its communistic regime, supported by the Soviet army, efficiently discouraged its citizens from developing and exhibiting creativity and excellence to maintain a culture of mediocrity and conformity. Universities were particularly affected in various ways: access to foreign scientific journals and textbooks was very limited; hermetic closure of the borders prevented students and scientists from traveling in either direction; and even basic chemicals and scientific instruments were difficult to obtain. Academic institutions were gray, uninspiring, and bureaucratic. Students rarely questioned their teachers, whose authority rested more on their position in the political hierarchy rather than on their knowledge and scientific achievement.

Luckily, there were exceptions though. In an unpublished essay from the late 1980s, the Slovak sociologists Martin Bútora, Vladimír Krivý, and Soňa Szomolányi coined the term “islands of positive deviation” which was also applicable to some academic departments led by scholars who managed to conduct excellent research and earn the respect of the international scientific community despite the unfavorable conditions they were working under. Some students, like me, by sheer luck, had a chance to become members of these groups during the most formative years of their lives. Not only were we able to work on interesting scientific projects under the supervision of an inspirational mentor. We were also exposed to the uplifting atmosphere of—sometimes semi-legal—seminars and vivid discussions not only about specific research projects, but also about scientific and cultural topics in general. For many of us, this liberal environment provided a glimpse of the academic freedom that existed elsewhere.

How was it possible to conduct competitive science under conditions that deprived us of even the most basic resources? No repressive system is completely airtight. We compensated for the lack of scientific journals by sending dozens of postcards to foreign scientists requesting reprints of their papers. When an envelope with a reprint—or occasionally an entire collection—arrived, we read every page eagerly and discussed it with our colleagues. The absence of Western textbooks was partly offset by poor-quality photocopies. And when someone eventually received permission to travel abroad to spend a few weeks in a foreign laboratory, they left with empty suitcases and returned with chemicals or instruments generously donated by our collaborators abroad. Because these resources were so difficult to obtain, they were highly valued and we used them with great care. Sometimes the lack of a reagent or appropriate infrastructure prompted unconventional experimental designs to address a particular research question. Scarcity encouraged deliberation, efficiency, creativity, and an appreciation of opportunities that others might have considered ordinary.

Forty years later, the situation is very different. Any scientific article is just a click away on our computer. If we need a chemical, it arrives at our bench the day after it was ordered. We can travel freely and attend scientific conferences all around the globe. Or watch the talks of prominent scientists online from our offices and not worry about tedious travel or tight budgets. A dream came true. We have gained access to formerly unavailable resources. We can spend our time more efficiently. But do we?

It seems to me that the instant availability of resources diminishes the value we assign to them. When obtaining a single scientific article required considerable effort, no further motivation was needed to read it carefully. Today, I download a pdf of an article, save it to a folder, and tell myself that I will read it later—but, too often I never do. When photocopying a rare book required hours of physical work, I read it from cover to cover. Now, I browse through a newly arrived hardcopy briefly and place it on a shelf for future consultation that rarely occurs. When a reagent costs a substantial portion of a laboratory’s annual budget and required months to acquire, experiments were planned carefully and discussed in every detail to make the best use of it. Today, express delivery does not necessarily improve outcomes. More than once, I have realized that an experiment conceived in haste was not worth pursuing to completion.

Hunger—in a general sense—is an unpleasant feeling signaling that we are deprived of our basic needs. But it also teaches us to appreciate what a well-fed person may take for granted. I am not suggesting that academia—or any society—should revert to the deprivation imposed by communist rule. Those conditions should never return. But there may be value in occasionally reminding ourselves—and our students—that abundance can lead to inefficiency and diminish our appreciation of readily available opportunities. A simple thought experiment—imagining how we would conduct our work if access to articles, books, reagents, or travel were severely restricted—may help us recognize opportunities more clearly and make better use of the extraordinary resources now available to us.

## Supplementary information

Peer Review File

