# Supplementary material for: The positive side of hunger
Source: EMBO Rep. 2026 Jul 18;27(15):4197–8. doi: 10.1038/s44319-026-00883-5 (PMC13458335; doi:10.1038/s44319-026-00883-5)
Supplement: Supplementary file 1 [file 44319_2026_883_MOESM1_ESM.pdf]

EMBO reports

The Positive Side of Hunger

Lubomir Tomaska

Corresponding author(s): Lubomir Tomaska (lubomir.tomaska@uniba.sk)

Review Timeline:

Submission Date:	9th Jul 26
Accepted:	13th Jul 26

Editor: Holger Breithaupt

Transaction Report: This article was editorially reviewed.
